# Supplementary material for: The Oocyte-Specific Linker Histone H1FOO Is Not Essential for Mouse Oogenesis and Fertility
Source: Cells. 2022 Nov 21;11(22):3706. doi: 10.3390/cells11223706 (PMC9688445; doi:10.3390/cells11223706)

Table S1. crRNAs and ssODN employed for the generation of the *H1foo* edited mouse model.

Nucleotides	Sequence (5′-3′)
crRNA1	GCAGGCCTTGACCGGGGCAG
crRNA2	AAGGCTGAAGAGCTGCCAGG
ssODN	GACAGTGGTCTGTGTAAGGCTTGCAGCCTAGCAGCTAGTGATGTCACTGGTTCTCAG-TGTGTGTGTGCTCTGCCCCACCAAAGCAGGCCTTGACCGGGGCGGGGGTGTGTCTCT CTCTGTATGGGAGGTCAGAAGAAATTCAAGGGGGTTTTGCACTAAGGAG-TCTGGGCTCATCCTAGAGCAGTGAGTAGCTATGAGAG

Table S2. Oligonucleotides employed for genotyping the mutant *H1foo* mice.

PCR (size)	Oligonucleotide name	Sequence (5′-3′)
WT (272 bp)	F1	ATGGAGAAAGGGCAGAAGAGG
	R1	CACTTCCTACGGAGGGAAACC
KO (268 bp)	F2	TGGTTCTCAGTGTGTGTGTGC
	R2	GCTGCCTTGAACTTCTGGTCT

Table S3. F2 breeding of a *H1foo*$^{-/-}$ male and two different *H1foo*$^{-/-}$ females showing an absence of a delayed phenotype.

Female ID	Number of litters	Mean of pups/litter
1	6	7,5
2	5	7

Figure S1. Double immunofluorescence of H1FOO (green) and SYCP3 (red) in spermatocytes showing an unspecific punctate staining in spermatogonia and a complete absence of H1FOO signal along prophase I (from leptotene to diplotene) and metaphase I in both WT and *H1foo*-deficient spermatocytes. Bars in panel, 10 μm.

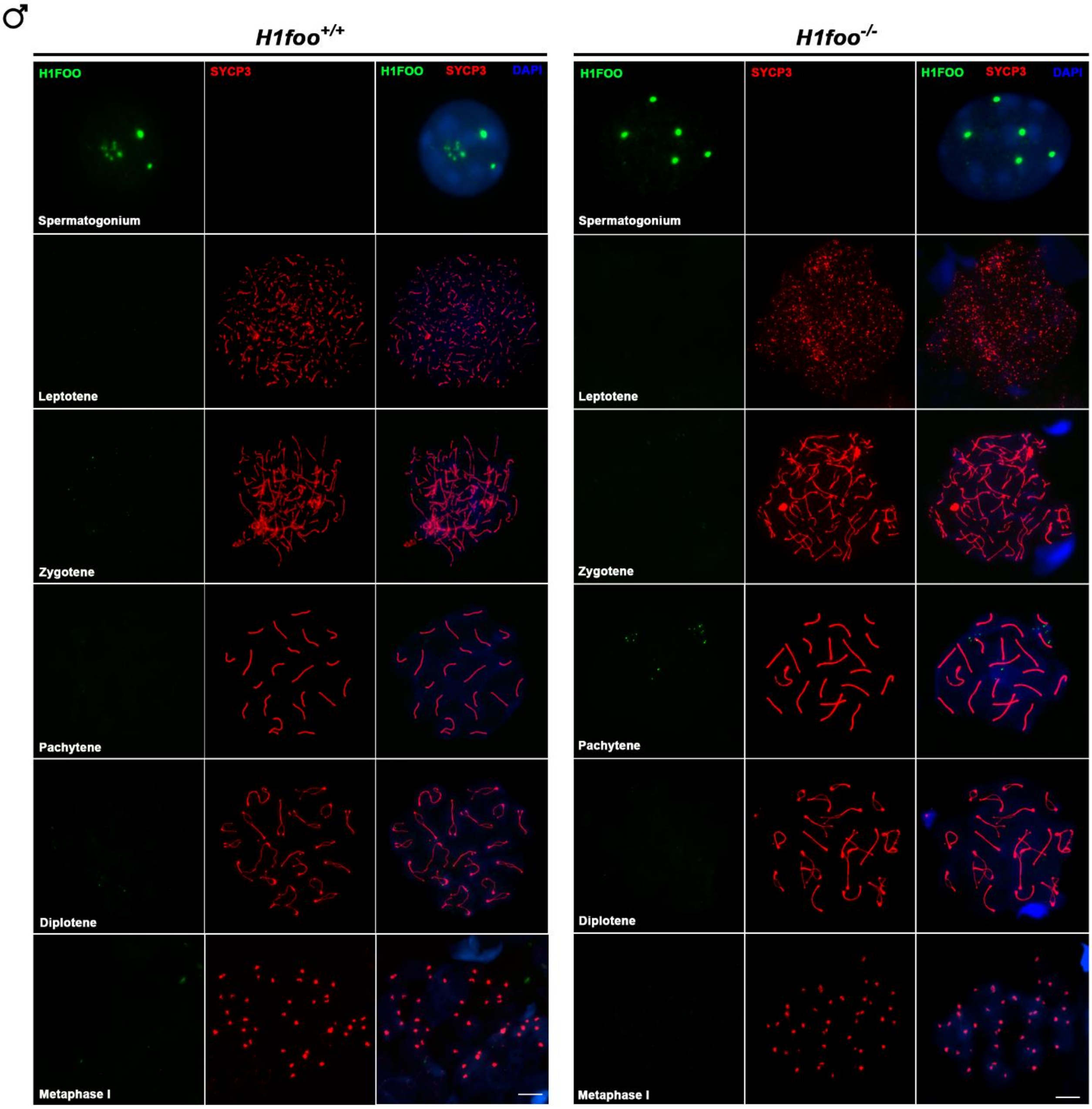

Figure S2. Double immunofluorescence of SYCP1 (green) and SYCP3 (red) in spermatocytes showing normal synapsis, desynapsis and progression of meiosis in WT and *H1foo*-deficient spermatocytes. Bars in panel, 10 μm.

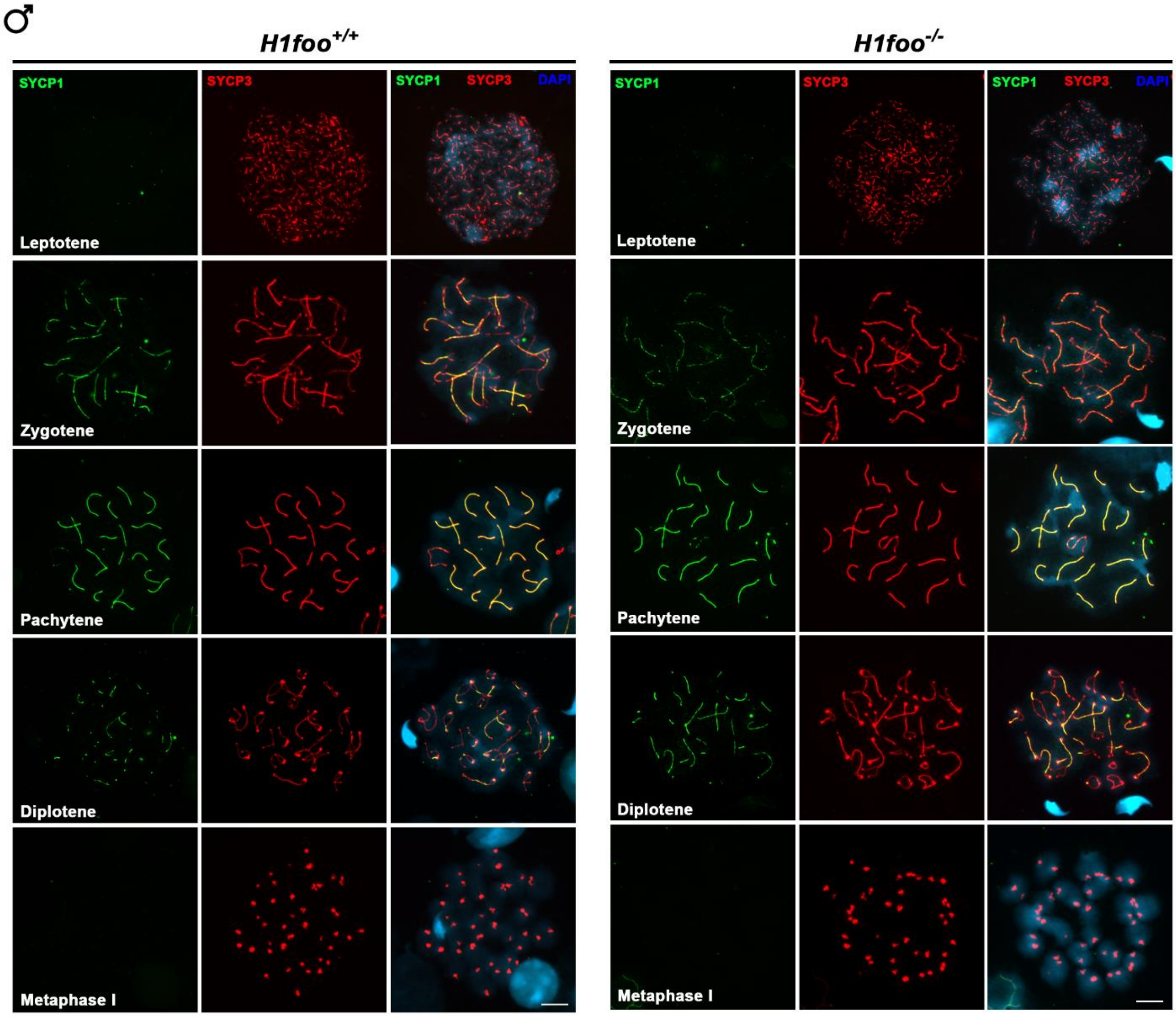

Figure S3. Double immunolabeling of SYCP1 (green) and SYCP3 (red) in 16.5 dpc oocytes. *H1foo*-deficient oocytes show proper chromosome behavior and synapsis/desynapsis processes demonstrated by the similar SYCP1 staining compared to their heterozygous counterparts. Plot under the panel displays the stage distribution analysis showing a faint, yet not significant delay of *H1foo* mutant oocytes at early prophase I. n=3. Two-tailed Welch's t-test analysis: n.s., no significant differences. Bars in panel, 10 μm.

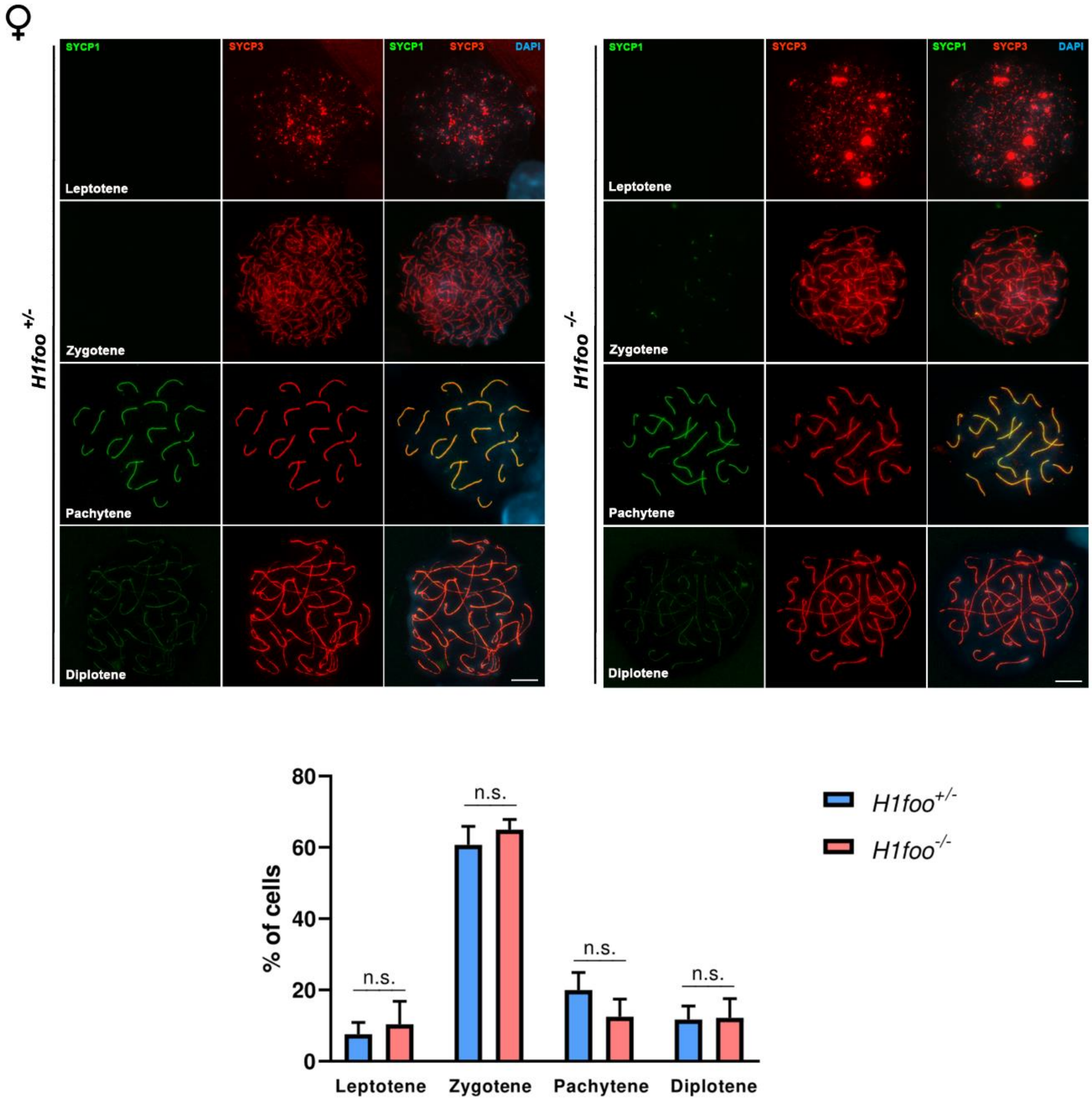

Figure S4. a) Double immunolabeling of γ-H2AX (green) and SYCP3 (red) in 16.5 dpc oocytes. Both *H1foo*-deficient and heterozygous oocytes show similar γ-H2AX distribution, as the signal appears decorating the entire nucleus at zygotene and subtly surrounding the chromosome axes at pachytene. Lower plot shows quantification of fluorescence signal at zygotene and pachytene stages. Two-tailed Welch's t-test analysis: n.s., no significant differences. **b)** Double immunolabeling of SUN1 (green) and SYCP3 (red) in 16.5 dpc oocytes showing properly regulated telomere dynamics in both heterozygous and *H1foo*-deficient zygonemas and pachynemas. **c)** Double immunostaining of ACA (red) and SYCP3 (green) evidencing proper organization of the centromeres in both heterozygous and *H1foo*-lacking zygonemas and pachynemas. Bars in panels (**a**-**c**), 10 μm.

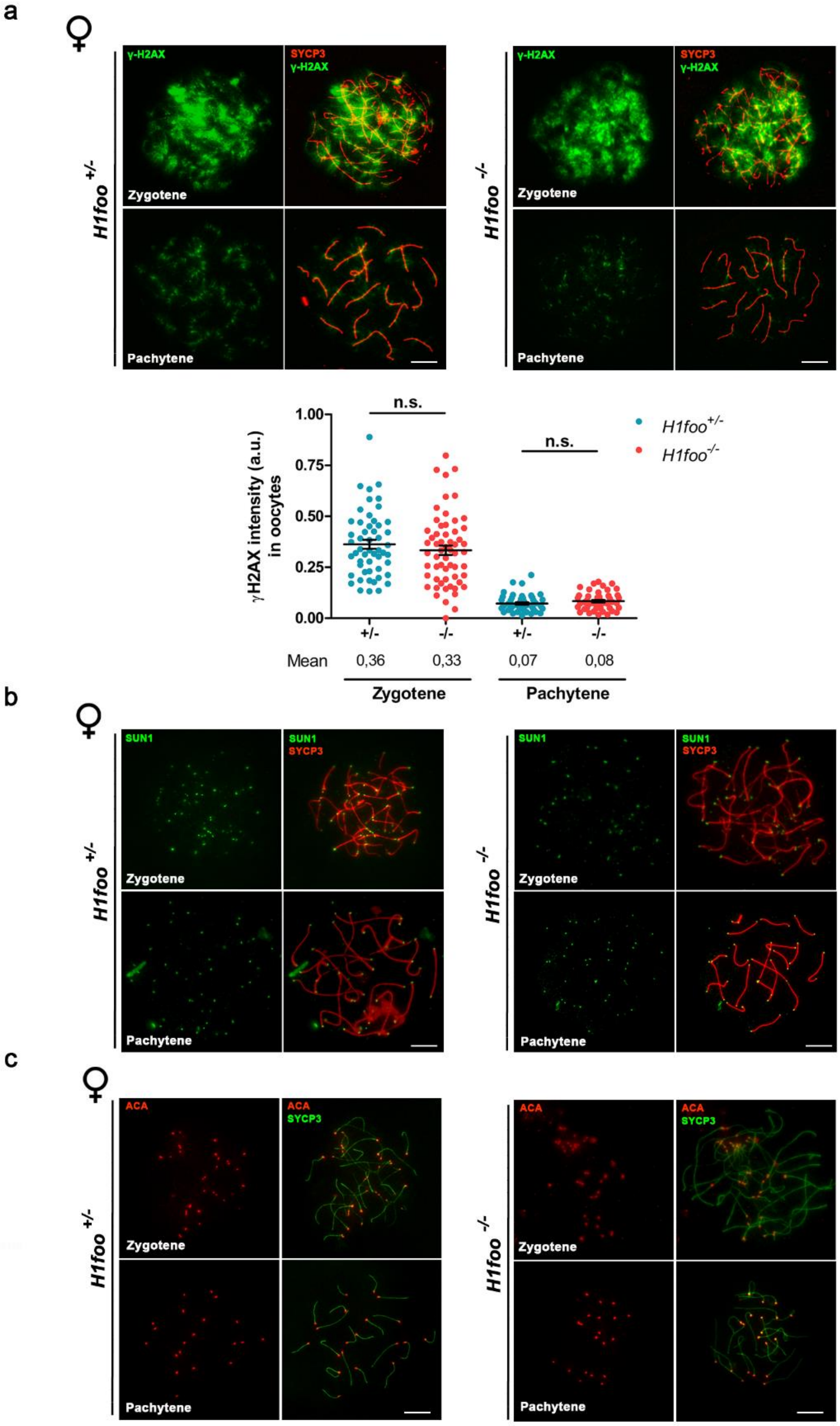

Supplement: Supplementary file 1 [file cells-11-03706-s001.zip › cells-2040569-supplementary.pdf]
